# The Remaining Challenge to Diagnose and Manage Cow’s Milk Allergy: An Opinion Paper to Daily Clinical Practice

**DOI:** 10.3390/nu15224762

**Published:** 2023-11-13

**Authors:** Yvan Vandenplas, Rosan Meyer, Anna Nowak-Wegrzyn, Silvia Salvatore, Carina Venter, Mario C. Vieira

**Affiliations:** 1 UZ Brussel, KidZ Health Castle, Vrije Universiteit Brussel (VUB), 1090 Brussels, Belgium; 2 Department Paediatrics, Imperial College London, London SW7 2BX, UK; 3 Department Dietetics, Winchester University, Winchester SO23 4NR, UK; 4 Department Medicine, KU Leuven, 3001 Leuven, Belgium; 5 Department of Pediatrics, NYU Grossman School of Medicine, Hassenfeld Children’s Hospital, New York, NY 10016, USA; 6 Department of Pediatrics, Gastroenterology and Nutrition, Collegium Medicum, University of Warmia and Mazury, 10-719 Olsztyn, Poland; 7 Department of Pediatrics, Hospital “F. Del Ponte”, University of Insubria, 21100 Varese, Italy; silvias.varese@gmail.com; 8 Section of Pediatric Allergy and Immunology, Children’s Hospital Colorado, University of Colorado, Aurora, CO 80045, USA; 9 Center for Pediatric Gastroenterology, Hospital Pequeno Príncipe, Curitiba 80250, Brazil; vieira.mcv@gmail.com

**Keywords:** cow’s milk allergy, IgE mediated, non-IgE mediated, prevalence, diagnosis, management, soy formula, hydrolyzed rice formula, extensive hydrolysate, amino acid formula

## Abstract

Guidelines and recommendations for the diagnosis and management of cow’s milk allergy (CMA) in childhood are based on scientific review of the available evidence. While this approach is the most rigorous, guidelines may not fully address all scenarios encountered by clinicians. Many symptoms of CMA overlap with other common childhood illnesses and are subjectively reported by the caregivers of the infant, as is the interpretation of the dietary interventions. Additionally, many healthcare professionals and caregivers do not follow the recommendations to perform an oral food challenge or reintroduction of cow’s milk after a diagnostic elimination diet because (1) the infant is doing well and (2) the carer’s fear of symptoms relapsing with this procedure. As a result, CMA in infants may be either under-diagnosed leading to reduced quality of life for families or over-diagnosed, resulting in unnecessary long-term elimination diets and increasing the risk for nutritional deficiencies. This paper discusses some of these controversial topics, focusing on misdiagnosis and mismanagement in clinical practice. The lack of objective diagnostic criteria can hamper the diagnosis and management of CMA in daily practice.

## 1. Introduction

Cow’s milk allergy (CMA) is the most common but also the most complex food allergy during infancy worldwide. The prevalence data, using a variety of diagnostic techniques, have primarily focused on immunoglobulin E (IgE)-mediated CMA and range from 1.8 to 7.5% in publications between 1973 and 2008 [1]. The 2023 position paper of the European Society of Pediatric Gastroenterology, Hepatology and Nutrition (ESPGHAN) reports that the prevalence of confirmed CMA is less than 1% [1].

The allergic journey (allergic march) frequently starts early in childhood. Hence, the primary aim should be the prevention of CMA. However, today there are no proven strategies for CMA prevention. Therefore, prevention will not be further discussed in this paper. When infants present with symptoms suggesting CMA, the health care professional (HCP) has the pivotal role of correctly diagnosing CMA, implementing the most appropriate management and supporting the timely development of tolerance.

The World Allergy Organization (Diagnosis and Rationale for Action against Cow’s Milk Allergy (DRACMA)), the European Academy of Allergology and Clinical Immunology (EAACI), the Global Allergy and Asthma European Network (GA^2^LEN), and the European Society of Pediatric Gastroenterology, Hepatology and Nutrition (ESPGHAN) are working on or recently published position papers/guidelines/recommendations on the management of CMA [1,2,3]. However, guidelines are often not followed because of the lack of awareness or difficulties with interpretation and application in daily practice.

The aim of this manuscript is to highlight and discuss a selection of topics that are still open for interpretation and debate on the diagnosis and management of CMA and to help HCPs in clinical practice.

## 2. Prevalence of Cow’s Milk Allergy

The prevalence of CMA ranges from less than 1% of the general population when documented with an oral food challenge (OFC), 1–4% according to most review papers, and up to 10% as perceived by caregivers and/or based on clinical suspicion [1,4,5]. In 2015, the incidence of CMA was estimated using the EuroPrevall birth cohort of 12,049 children from across Europe, which included both IgE- and non-IgE-mediated allergies. The overall challenge-proven diagnosis was 0.54%, but there was great variation in the reported incidence of non-IgE-mediated allergy across countries. In the United Kingdom, more children presented with non-IgE-mediated CMA (56.3%) than IgE-mediated CMA (43.7%), while the prevalence of non-IgE-mediated CMA was not reported in many countries, including Spain, Lithuania, Greece and Germany [6]. According to a very recent updated systematic review and meta-analysis, the overall pooled estimate for all age groups of self-reported lifetime prevalence for cow’s milk (CM) was 5.7% (95% confidence interval (CI) 4.4–6.9), while the point prevalence of food-challenge-verified allergy for CM was 0.3%, (0.1–0.5) [7].

In the United States, Food Protein-Induced Enterocolitis Syndrome (FPIES) is estimated to occur in 0.51% of <18-year-old subjects [8], and Food Protein-Induced Allergic Proctocolitis (FPIAP) may occur in up to 17% of infants based on pediatrician diagnosis, though these estimates were not confirmed by an oral food challenge (OFC) [9]. A systematic review including data from 15 countries across the five continents indicated a world-wide incidence of eosinophilic esophagitis (EoE) of 4.95 cases per 100,000 inhabitant years [10]. CMA is the most common food allergen in these presentations of non-IgE-mediated food allergies. There is an accumulating evidence that non-IgE CMA is a risk factor for IgE CMA and peanut allergy [11].

## 3. Symptoms and Diagnosis

**Figure 1:**
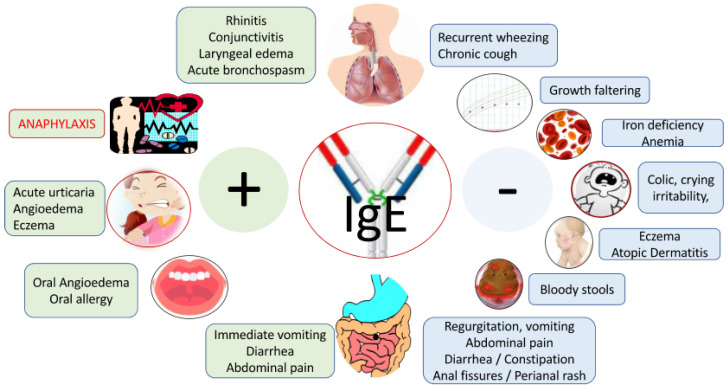
Signs and symptoms associated with cow’s milk allergy (adapted from Ref. [1]). Legend: patients may also present with mixed IgE and non-IgE CMA symptoms; none of the symptoms are specific; symptoms are unrelated to infection.

All symptoms of CMA, including anaphylaxis, are non-specific, and can be caused by a spectrum of different diseases or other allergies, which makes CMA diagnosis challenging. The variety of symptoms are extensively discussed in many papers and are listed here but will not be further elaborated in detail (Figure 1: symptoms of CMA) [1,2].

The duration of the recommended diagnostic elimination diet differs for IgE- and non-IgE-mediated allergy: 1–2 weeks for IgE-mediated and 2–4 weeks for non-IgE-mediated allergy, and 8–12 weeks for EoE [1]. The literature is not clear if this is for “significant improvement” of the symptoms or “symptom resolution”. The gold standard diagnostic tool for IgE- and non-IgE-mediated CMA is a double-blind placebo-controlled food challenge (DBPCFC), typically utilized in clinical trials of therapies for IgE-mediated food allergy [1]. However, as DBPCFCs are complex, laborious, and expensive, they are extremely difficult to perform in daily practice. Therefore, an open oral food challenge (OFC) is an acceptable alternative in most recommendations and guidelines for clinical practice [1,12].

The interpretation of the OFC outcome or reintroduction is the responsibility of a clinician with experience in food allergy. The reintroduction can be performed at home in cases of non-IgE-mediated allergy (except for food protein-induced enterocolitis syndrome (FPIES)), while in case of IgE-mediated allergy, the OFC should be performed under direct medical supervision, in a hospital or office setting because of methodological issues and potential severe reactions. Practical OFC guidelines provide a blueprint for standardizing OFC and are used widely in research studies for IgE-mediated food allergy [13]. Alternative standardized protocols on “how to perform a challenge” have been proposed [14,15]. There are, however, no trials directly comparing different protocols, so it is not known which OFC protocol performs best. The in-hospital OFC lasts usually half a day, including to perform the test, to observe the early phase of reintroduction and to treat potentially severe (mostly IgE-mediated) clinical manifestations, while reactions (mainly non-IgE-mediated) may occur up to one week after the introduction of CM in the diet. If the supervised OFC is negative (no observed allergic symptoms), CM should be offered to the infant at least ~250 mL/day or an age-appropriate portion of milk-based products during the following week at home, tracking the potential reappearance of symptoms. The prolonged observation is always necessary to also exclude non-IgE-mediated CMA as some patients can suffer from both conditions simultaneously. In non-IgE-CMA the interpretation of the effect of the reintroduction of CM in the infant’s diet is based on the subjective parental reporting. Considering a non-specific nature of the delayed symptoms, e.g., skin rashes, vomiting, loose stools or discomfort, there is a significant risk of over-interpreting these at-home OFCs as positive. Ideally, the at-home OFC should be performed in a blinded manner with a recording of symptoms at baseline and during the at-home OFC, however this is logistically difficult to execute and thus rarely performed in clinical practice.

The complicating factor is the frequent refusal to challenge or reintroduce the food by parents. This may be influenced by differences in insurance coverage and health care systems, the reimbursement of infant formulas and/or the availability and accessibility of HCPs or the unwillingness of the parents to manage the returning symptoms. Data on the prevalence of this refusal are limited. In one clinical trial, 23% of caregivers declined an OFC in a randomized controlled trial where both parents had to sign an informed consent that a challenge test had to be performed as part of the trial [16].

As a consequence, the true prevalence of non-IgE-mediated CMA outside of research studies can only be estimated and not proven. Infants of parents who refuse an OFC or reintroduction are considered by the “scientific world” as non-allergic since there has been no definitive proof of allergy. Thus, by requiring these steps to confirm the diagnosis, the prevalence might be underestimated. However, in the absence of the clinical documented relapse at reintroduction of CM in the diet, CMA will be definitely over-diagnosed. This has potential detrimental consequences for infant nutrition and increased cost of hypoallergenic infant formulas when not breastfed, incurred both by the health systems and by the families. There is a need for educating parents of the importance of the OFC following a trial of a diagnostic elimination diet and for establishing clear criteria when to embark on such diagnostic diet for non-IgE-CMA [1].

### 3.1. IgE-Mediated CMA

The measurement of CM-specific IgE and skin prick tests are useful in the diagnosis of IgE-mediated reactions, although they merely confirm the IgE-sensitization and not a clinical allergy. False positive tests are common, especially with a small size of the skin prick test wheal and low serum food-specific IgE level. The higher the serum food-specific IgE level, the lower the likelihood of the acquisition of tolerance at a younger age [17]. Overall, IgE-mediated CMA persists longer than non-IgE-CMA [18]. When an anaphylactic reaction has been recently reported or when specific IgE to CM or individual CM proteins (e.g., casein, alpha-lactalbumin, beta-lactoglobulin) are elevated, the OFC should either be deferred or performed in a controlled setting. However, the cut-off limits of specific IgE to predict a clinical allergy in an individual patient and to eliminate the need for the CM OFC are population-specific and not universally endorsed.

### 3.2. Non-IgE-mediated CMA

In non-IgE CMA, it is technically challenging to perform the reintroduction of CM in a double-blind manner because it may take several days before the relapse of symptoms, although this has been performed in study context [19]. The blinding of the home challenge is theoretically possible but very difficult in clinical practice, as it requires resources to provide specific foods and medication for home, parental adherence and reliable reporting of symptoms. This means that the HCP will always have to rely on the information provided by the parents in case the reintroduction was performed/continued at home [20].

The lack of a specific symptom of non-IgE CMA hinders the clinical distinction. Since 25–50% of infants present with a disorder of gut–brain interaction (DGBI), which is often considered to be related to milk intake, CMA is often suspected as potential diagnosis [21,22]. The only diagnostic tool for non-IgE-mediated allergy is an elimination diet for 2–4 weeks (or 8–12 weeks in EoE), during which symptoms should improve followed by a relapse after the reintroduction of CM. However, the clinical improvement and reappearance of symptoms “on” and “off” the diet may occur also in a DGBI but due to different pathophysiologic factor (i.e., motility, fermentation) and not to immunologic mechanisms, hampering a correct diagnosis.

### 3.3. Disorders of Gut–Brain Interaction (DGBI) and CMA

In many infants, non-IgE-CMA and DGBI cannot be clearly distinguished from each other due to the similarity of symptoms, and both can present with symptoms related to exposure to CM. DGBI, formerly called functional gastro-intestinal disorder (FGID), of which symptoms include constipation, regurgitation, and infant colic, are often considered as a normal developmental stage during infancy not needing intervention. A population-based estimate suggests that up to 25% of children up to 5 years had at least three consultations with an HCP because of gastro-esophageal reflux disease (GERD) symptoms [23]. GERD in infants is non-specific, and the differentiation between GER and GERD is difficult in regard to medication management. Pharmacological treatment with gastric acid inhibitors and prokinetics are widely used without objective investigations to guide treatment [24]. The evidence to support the efficacy and safety of H^2^ receptor antagonists (H^2^RAs) in infants and children are limited and of poor quality. Furthermore, H^2^RAs do not reduce crying/distress and visible regurgitation/vomiting in children with GERD compared to the placebo [25]. Ranitidine was the only H^2^RA studied in infants and children, and thus, it is the most commonly used compound for GERD but has been taken off the market because of the presence of nitrosamines. There is insufficient evidence to recommend the routine use of proton pump inhibitors (PPI) for infants <1 year of age with FA-associated GERD. PPIs have a significant impact on selected micronutrient availability, one’s microbiome, the risk of developing infections, food allergy and protein breakdown and require special attention to counteract these effects [26].

However, it is well accepted that similar symptoms that affect quality of life (QoL) in adults should be managed with interventions to improve the subject’s feeling of well-being [27]. It should be recognized that QoL is also of importance for infants and their families but is insufficiently investigated and considered in the pediatric age group.

The differential diagnosis between non-IgE-mediated allergy and a DGBI remains probably one of the most challenging issues [1]. If an infant with a DGBI, also presents with atopic dermatitis and/or respiratory tract symptoms, and this improves during a diagnostic elimination diet, non-IgE-mediated allergy is likely because of a shared immune-mediated and motility-mediated pathophysiologic mechanism. Noteworthy, a patient may also present with a combination of disorders, i.e., non-IgE-mediated CMA and DGBI, as one condition does not exclude the other.

The major challenge is when the symptomatology is restricted to the GI tract (regurgitation, vomiting, reflux, constipation or diarrhea) and general symptoms (crying and distress). In both situations (DGBI or non-IgE CMA), the vast majority of the infants present with a combination of these symptoms [22]. There is a large overlap between normal healthy physiology and mild pathology. Every infant cries or is distressed for a certain amount of time during the day [28,29]. At least half of the infants do regurgitate regularly in the first months of life and only a minority of them will suffer from CMA or GERD. It is a challenge for an HCP to identify the pathophysiologic mechanism for the crying, regurgitation and vomiting. To help the clinician’s decision process and improve patient’s care, a pragmatic stepwise approach has been proposed [22].

Parental perception of stool consistency and frequency, regurgitation, crying and distress and accessibility of health care will determine what is tolerated and how fast HCPs are consulted. The estimation of the severity of DGBI is always biased as it is fully dependent on the description by the caregivers. The use of artificial intelligence may in the near future result in more objective reporting of stool consistency [30]. Recently, the difference in the perception of normality in infant distress and crying was observed when a standardized tool of CM-related symptom score (CoMiSS^TM^) was tested in presumed healthy infants [29]. The tolerance for crying perceived as normal behavior was much larger in Poland than in Belgium and Italy [29]. The same was observed for regurgitation in Indonesia compared with Belgium [31]. Many HCPs may over-consider CMA as a possible diagnosis since the symptoms are “feeding” related. Nonetheless, although many of these infants present with symptoms that can be regarded as within the spectrum of physiology, there is an associated decreased QoL of the family and the infant [32]. Therefore, parental reassurance and anticipatory guidance regarding the natural development of these symptoms is recommended. A detailed diary should be recommended to the caregivers in order to identify and track symptoms. It is well known that a prospective diary for crying time suggests much less crying than spontaneously and retrospectively reported by parents. We reported similar experience for regurgitation [33]. The QoL of the family and infant should also be at the forefront of every decision by the HCP.

For both CMA and DGBI, suspected or documented CMA is not a reason to stop breastfeeding. Breastfeeding should be supported in these circumstances, and it may be beneficial to obtain advice of a breastfeeding specialist regarding the latch and feeding techniques. “Comfort formulas” are positioned as add-on management for mixed- or formula-fed infants with a DGBI since there are data suggesting some efficacy and because these formulas are generally safe and well tolerated [34]. Although some studies with comfort formulas were randomized and blinded, it is almost impossible to effectively blind formula trials because of differences in texture (thickening), smell and taste. Many infants may improve with comfort formulas because of a placebo effect, which is reported to be up to 20%. Most of these formulas contain partially hydrolyzed whey protein as a protein source, are thickened and have adaptations to decrease stool consistency (high magnesium, pre- or probiotics, palmitic acid in the sn-2 position). While pHFs are not recommended as hypoallergenic options in the management of CMA, up to 50% of infants with a positive DBPCFC, confirming the diagnosis of CMA, may also tolerate a pHF [35]. Thus, improvement with a pHF does not exclude the possibility of either CMA or DGBIs.

The question arises whether it is important to distinguish between DGBI and non-IgE CMA or if they could be considered as being part of the same CMA spectrum disorder, which can benefit from the same management? An important consideration for the above question is that children with non-IgE mediated CMA are at higher risk of developing other allergic manifestations, including atopic dermatitis, IgE-mediated food allergy, asthma, and allergic rhinitis, and accurate diagnosis informs anticipatory guidance [11,36].

## 4. Awareness Tools

Several “awareness tools” have been developed to improve the recognition of CMA. Among them, the Cow’s Milk-Related Symptom Score (CoMiSS^TM^) is the most extensively studied. Initially, CoMiSS was developed to simplify the reporting of symptoms of CMA at inclusion and their evolution during a clinical trial [16]. Later, CoMiSS was proposed as a tool to raise awareness of HCPs that symptoms such as crying/distress, regurgitation/vomiting dermatitis, respiratory manifestations and stool pattern may be related to milk intake and, particularly the possibility of CMA. Particularly, above a certain CoMiSS score (≥10), the HCP should consider CMA as possibly responsible for the clinical findings [37]. It is hotly debated if such awareness tools lead to the over-diagnosis of CMA. As a result, a group of experts have reviewed the data for all tools (including online questionnaires and diagnostic tools) that are available in English language and have developed Delphi consensus statements to support the appropriate use of these tools [38]. The authors recommend that questionnaires/tools should be validated prior to clinical application and should only be used with the involvement of an HCP with knowledge of food allergy to establish the relevance of the symptoms in order to avoid the over-diagnosis of food allergy when common gastrointestinal manifestations are present.

Although over-diagnosis due to increased awareness by using tools such as CoMiSS cannot be excluded, it is also difficult to prove that the latter is effectively the case given the fact that there are no other diagnostic tools available in non-IgE CMA allergy than elimination diet and the reintroduction of CM. It is only when the reintroduction causes symptoms that the diagnosis of CMA can be confirmed.

HCPs and parents often consider isolated DGBI manifestations such as colic (crying and distress), regurgitation or vomiting, and defecation difficulties (constipation or diarrhea) as symptoms of CMA. The use of an awareness tool like CoMiSS necessitates the presence of a minimum two of these symptoms to be possibly indicative for CMA, suggesting that the application of an awareness tool might as well decrease the prevalence of the suspicion of CMA. A shortcoming of the symptomatology of CMA, and of the symptoms included in CoMiSS, is that they rely on parental reporting, and thus are prone to subjective interpretation. Efforts were made to make at least the report of the stool consistency more objective, since artificial intelligence based on an algorithm of thousands of photos of stools made the stool composition interpretation more objective [30].

## 5. Therapeutic Elimination Diet

**Figure 2:**
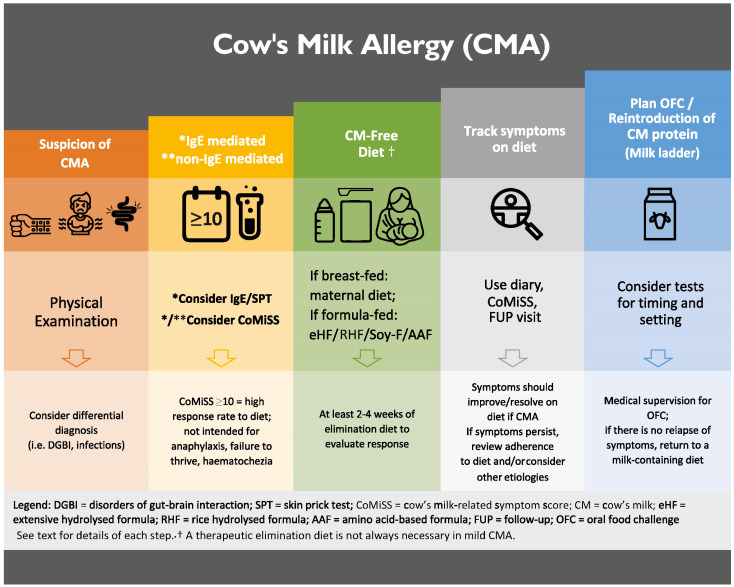
Practical management in infants suspected to suffer CMA. * Refers to each other: consider IgE/SPT and CoMiSS is for IgE mediated allergy. ** consider CoMiSS is only for non-IgE mediated allergy.

The goals of CMA management are three-fold: (i) the resolution of symptoms, (ii) the acquisition of tolerance to CM, and (iii) supporting normal growth and development. The practical approach is illustrated in Figure 2.

Currently, there is no curative therapy for CMA except for oral immunotherapy (OIT) that, in some patients, might induce tolerance. For the majority of children with CMA, standard management relies on the avoidance of the CM protein. Breastmilk is the best source of nutrition for all infants and should be promoted as the first-line management option in all infants that are breastfed, including the ones with CMA. When breastmilk is not available, most guidelines recommend a CM-based extensive hydrolysate (eHF) as the first-choice option for formula-fed infants with mild to moderate symptoms, as the peptides present in these special formulas are tolerated by at least 90% of all CMA infants. A minority of infants may react to the eHF, as it still contains residual CM peptides [1]. The failure to tolerate eHF is estimated at ~5%, although a prevalence of up to 20% (even 50%) for some eHFs has been reported [39,40]. However, these high drop-out rates are observational and mostly derived from suspected non-IgE CMA and thus confounded by a potential over-diagnosis. Hydrolyzed rice formulas (HRFs) are increasingly being utilized since they are free of CM proteins, similar to amino acid-based formulas (AAFs) [41].

An important consideration regarding the unwarranted use of therapeutic infant formulas is that due to the hydrolysis of CM proteins and the presence of amino acids, as they have a different taste and have been shown to have a potential long-term impact on taste preferences [42]. It remains to be determined whether different taste preferences have significant health consequences.

### 5.1. Hypoallergenic Formula Options for Non-Breastfed Infants

Currently, in severe CMA (including growth-faltering or anaphylaxis), all guidelines recommend that an AAF should be used as the first-line feed in the absence of breastmilk [1]. There has been the suggestion to use an AAF as a first-line diagnostic elimination diet for all formula fed infants suspected to suffer from CMA, which would solve the issue of intolerance to the eHF at diagnosis [43,44,45]. However, AAF is much more expensive than eHF and a diagnostic OFC is critical to justify the need for AAF as a therapeutic elimination diet. Since there is often a reluctance to perform a diagnostic OFC or reintroduce CM and OFC services are often limited and have long waiting lists, this approach would likely result in an overuse of this expensive feed, despite it being nutritionally adequate.

Soy infant formula has always been an option for the management of CMA, since it is also CM-free and less expensive than eHF. The concerns for the potential adverse effects of phytoestrogens and isoflavones have hampered the use of soy formula below 6 months of age. The modern soy formulas are nutritionally adapted for infants, and the concerns regarding phytoestrogens and isoflavones are no longer valid [1,46]. However, the prevalence of soy allergy is debated and not adequately investigated; in some guidelines it seems to be comparable to that of CM [1]. A systematic review with meta-analysis including 40 studies published from 1909 to 2013, identified the established weighted prevalence of soy allergy as 0 to 0.5% (0.27) for the general population, 0.4 to 3.1% (1.9) for the referral population, and 0 to 12.9% (2.7) for the allergic children [47]. The prevalence of IgE sensitization after the use of soy-based formulas is around 8.7 [47]. Thus, soy infant formula might result in as many allergic infants as using CM formula, although with an allergy to a different protein. The data on non-IgE-mediated CMA and on soya allergy have primarily been reliant on retrospective and observational reviews. According to these, up to 50% of children with a non-IgE-mediated CMA may also react to soy [48,49,50]. However, geographic differences in concomitant soy allergy have been found in particular when comparing common triggers for FPIES [51].

Over the past two decades, hydrolyzed rice-based formulas (HRFs) have been increasingly available in Europe, Asia, Central and Latin America, and the Middle East. Although less well studied than CM-based eHFs, HRFs are considered to be nutritionally safe [1,52]. The four published randomized trials show a 100% efficacy in the management of CMA [53,54,55,56]. RHFs are 100% CM-free. Therefore, the recent DRACMA guidelines and ESPGHAN position paper consider them as an “alternative” to eHFs [1,52]. It has been suggested that in the future, with more evidence supporting their safety and nutritional value, HRFs might become the first-choice option for CMA management [52]. HRFs are also shown to be nutritionally adequate and to result in normal growth. Since allergy to rice is rare, studies comparing the efficacy of partially hydrolyzed rice formula and intact rice-protein-based formula are desirable. Data are also missing regarding the efficacy of HRFs, specifically in CMA-infants not tolerating eHF. Due to the concerns about the high levels of arsenic in rice, the arsenic content of the HRFs should always be measured and disclosed on the label [1]. However, the arsenic content in HRFs is lower than in CM-based formula, and (tap) water is likely to be the most important source of arsenic in formula [57].

There has been a debate related to lactose as an ingredient in eHF. The primary carbohydrate source in human milk is lactose, and therefore lactose is the recommended carbohydrate in infant formula. Historically, lactose in infant formula was manufactured from CM and was excluded from the hypoallergenic formulas launched for CMA due to concerns about contamination with small amounts of CM protein. However, technology has allowed for industrially produced lactose to be 100% CM free. Although it seems logical to prefer lactose over other carbohydrates, no clinically relevant adverse effects of lactose-free formulas have been demonstrated. If diarrhea is a major presenting symptom of CMA, a time-limited lactose-free diet may be considered because there may be a transient lactase deficiency. Unfortunately, the time required for the gastro-intestinal tract to recover is not known [1].

### 5.2. Therapeutic Elimination Diet Is Not Always Necessary in Mild CMA

Mild symptoms of gastroesophageal reflux, intermittent rectal bleeding, abdominal discomfort, or eczema skin rashes do not necessitate dietary elimination. These symptoms can be managed medically, e.g., with positioning and burping after feeding, using skin moisturizers and mild potency topical corticosteroids for eczema. Mild intermittent rectal bleeding in FPIAP may be observed without any dietary interventions, following a discussion with the parents regarding potential risks and benefits, and shared decision on management [1]. If symptoms are severe or worsening or the infant is not thriving, maternal diet can be modified to eliminate CM protein; in a non-breastfed infant, a hypoallergenic formula is introduced. Alternative sources of nutrients, calcium and vitamin D should be provided to replace CM and dairy products in maternal diet.

### 5.3. “Biotics” in Infant Formula

Many formulas for CMA management (and prevention) are supplemented with various “biotics”: probiotics, prebiotics (including HMO-’analogues’) or synbiotics (a combination of pre- and probiotics). There is convincing evidence that the addition of these “biotics” has an impact on the gastro-intestinal microbiota composition. While subtle differences are reported in the effect of each biotic, overall, they mainly increase the abundance of bifidobacteria, increase short-chain fatty acids, reduce the pH and decrease potential pathogens. They bring the gastro-intestinal microbiota composition of these formula-fed infants closer to that of breastfed infants [58,59,60]. Secondary outcomes suggest an immunomodulatory effect, resulting in a decrease in inflammation, infections, and the prescription of antibiotics [61,62]. However, additional benefits on the efficacy of hydrolysates with added probiotics in the management of CMA have not been demonstrated [1]. It is also unclear if these biotics enhance the development of oral tolerance, although this has been suggested for *Lacticaseibacillus* (L.) *rhamnosus* GG [1]. LGG may have moderate-quality evidence to promote oral tolerance in children with CMA and may facilitate recovery from intestinal symptoms [63]. However, this finding must be treated with caution, as more RCTs are needed to evaluate the most effective dose and treatment time for children with CMA independent from company sponsorship [63]. The literature reports as well a slower and more rapid acquisition of oral tolerance for CM when comparing RHFs and CM eHFs [55,64].

## 6. Natural History of CMA and Strategies for Reintroduction of Cow’s Milk

The natural history of CMA is generally very favorable, with the majority of children achieving tolerance by age 3 years [6]. However, CMA is a part of an atopic march and can be followed by wheezing/asthma and allergic rhinitis, with these conditions developing simultaneously with or subsequent to CMA. The timing of CM reintroduction following a therapeutic elimination diet is based on the type of allergy, severity, and age. Unnecessary prolonged elimination diets should be avoided and the proactive management of CMA is recommended.

(A) Infants and young children younger than 3 years

In general, mild non-IgE-mediated forms of CMA in infancy resolve sooner than IgE-mediated CMA, since according to the EuroPrevall data all infants with non-IgE mediated CMA were CM tolerant at the age of one year [6]. As such, in FPIAP, CM reintroduction can be attempted after age 6 months and repeated, if needed, periodically, e.g., every 2–3 months until tolerance is established. In FPIES, a minimum 6 months since the most recent reaction before reintroduction is advisable, with a typical period of avoidance between 12–24 months [65]. In IgE-mediated CMA, the timing of reintroduction can be guided by the monitoring of SPT and serum CM-specific IgE levels every 6–12 months, with decreasing values indicating favorable likelihood of tolerance [2,51,66]. Since the majority of children with CMA tolerate milk in the form of baked products, the reintroduction may be initiated with baked foods. Importantly, children who react to baked milk have more severe (higher risk of anaphylaxis) and more persistent milk allergy, therefore the attempts at reintroduction are safest when performed under physician supervision [67]. In settings where access to specialists and resources is limited, gradual home reintroduction may be considered in infants and young children under age 3 without prior anaphylaxis and no history of wheezing illness [68]. Recently a group in Ireland has used the MAP ladder for the introduction of CM in 60 infants with IgE-mediated CMA, mean skin prick test of 5.96 mm and CM-specific IgE of 11.3 kU/L, starting at a mean age of 7.3 months, following a passed supervised challenge to 0.5 mg CM protein (approximately 0.015 mL of liquid milk), which is the eliciting dose ED05 established for 5% of patients with CMA [69]. There were three serious adverse events reported: one child had acute viral laryngotracheobronchitis, unrelated to the study, and two children had reactions to milk at steps above their current tolerated level on the milk ladder. At 12 months, 64% of the children following this approach, versus 37% of the standard avoidance diet advice, were fully tolerant of CM (*p* < 0.05) [69].

If milk ladders are used, they should preferably be standardized and adapted to local dietary habits [70]. In case the child is still intolerant, or if the OFC is postponed, there is consensus to re-test every 6 to 12 months, although the best time-frame has never been studied [1,51]. In daily life, many infants have CM protein accidentally introduced in their diet by caregivers by ignorance, revealing their state of persistent allergy or acquired tolerance.

(B) Children older than 3 years

In both IgE- and non-IgE mediated CMA, persistence beyond age 3 years is usually associated with a more persistent and possibly more severe forms of CMA [71]. The timing of follow up evaluations and reintroduction attempts in non-IgE CMA is typically a shared decision with the caregivers; FPIAP and FPE to CM can be re-trialed at home every 6–12 months or more frequently if symptoms are mild. In FPIES, attempts at reintroduction can be repeated under physician supervision, usually every 12–18 months, or sooner, if the most recent symptoms were mild.

In IgE-mediated CMA, children reactive to baked milk, those with peak life-time CM-sIgE > 50 or severe atopic dermatitis are likely to have a more prolonged CMA. In one approach, CM-sIgE levels and SPT are repeated every 12 months, and the timing of the reintroduction attempts is based on the magnitude of decrease in these parameters. A CM-sIgE decrease by 50% over 24 months has been proposed as a favorable indicator of the increased likelihood of tolerance [72].

## 7. Conclusions

Breastfeeding is the first-choice method infant feeding. IgE- and non-IgE-mediated CMA, including acute FPIES, can occur in exclusively breastfed infants. Overall, the prevalence of confirmed IgE-mediated CMA is less than 1%, equally in breastfed and in formula-fed infants. However, the prevalence of non-IgE-mediated CMA is debated because of the non-specific symptoms and the lack of a diagnostic biomarker. Currently, the reintroduction of CM in the diet (challenge) after the resolution of the symptoms on a CM free diet is considered as the best diagnostic test. However, this is often declined by the family. The lack of the confirmatory challenge causes the over-diagnosis of CMA with a prolonged unnecessary elimination diet, and parents need to be informed about the benefits of the challenge and the risks of the unnecessary dietary restrictions from the beginning of the diagnostic journey. Conversely, the lack of awareness of CMA will result in under-diagnosis. In order to mitigate under-diagnosis, CMA awareness tools were developed but do not replace clinical judgement and diagnostic tests, where appropriate. The therapeutic elimination diet can be CM-based eHF or HRF. At this moment, AAF is indicated in infants with life-threatening conditions or growth-faltering, including anaphylaxis, EoE and severe FPIES. If more data are available, HRF may be proposed as an alternative for AAF.

The addition of “biotics” stimulates the growth of bifidobacteria and reduces frequency of infections. There is insufficient evidence to recommend any intervention to enhance the development of tolerance to CM.

## Abbreviations

AAF: Amino acid formula; CM: cow’s milk; CMA: cow’s milk allergy; CoMiSS: cow’s milk-related symptom score; DGBI: disorders of gut–brain interaction; DBPCFC: double-blind placebo controlled food challenge; EAACI: European Academy of Allergology and Clinical Immunology; eHF: extensively hydrolyzed formula; EoE: eosinophilic esophagitis; FPIAP: food protein-induced allergic proctocolitis; ESPGHAN: European Society of Pediatric Gastroenterology, Hepatology and Nutrition; FPIES: food protein-induced enterocolitis syndrome; GER(D): gastro-esophageal reflux (disease); HCP: health care professional; H^2^RA: hydrogen receptor antagonist; HRF: hydrolyzed rice formula; IgE: immunoglobulin E; OFC: oral food challenge; OIT: oral immune therapy; pHF: partial hydrolyzed formula; PPI: proton pump inhibitor; QoL: quality of life.
